# Firefighter Stress, Anxiety, and Diminished Compliance-Oriented Safety Behaviors: Consequences of Passive Safety Leadership in the Fire Service?

**DOI:** 10.3390/fire6060241

**Published:** 2023-06-18

**Authors:** Todd D. Smith, Mari-Amanda Dyal, David M. DeJoy

**Affiliations:** 1 Department of Applied Health Science, Indiana University School of Public Health–Bloomington, Bloomington, IN 47405, USA; 2 Department of Health Promotion and Physical Education, Kennesaw State University, Kennesaw, GA 30144, USA; 3 Department of Health Promotion and Behavior, College of Public Health, University of Georgia, Athens, GA 30602, USA

**Keywords:** firefighter, safety behavior, stress, anxiety, personal protective equipment, leadership

## Abstract

Safety-specific passive leadership has been negatively linked to diminished safety outcomes, including safety behaviors. However, this relationship is not fully understood. Research has not fully examined mediating factors that may be influenced by passive leadership, which then influence safety behaviors. Research among firefighters in this context is particularly absent. As such, this study aimed to examine relationships between safety-specific passive leadership, stress, anxiety, and compliance-oriented safety behavior outcomes among 708 professional firefighters. A path analysis was completed. The hypothesized model fit was very good and hypothesized relationships were confirmed. Safety-specific passive leadership was positively, significantly associated with increased firefighter stress perceptions and stress was positively, significantly associated with anxiety. Anxiety was negatively, significantly associated with both safety compliance and personal protective equipment behavior. This study has implications for researchers and practitioners. The findings emphasize the importance of active leaders in the fire service as passive leadership in the context of safety is distressing, which results in anxiety and ultimately diminished safety behavior outcomes, which could place firefighters at risk for injuries, illness, or death.

## 1. Introduction

That poor leadership negatively impacts individuals is not new [1,2]; however, there is still much to learn about the negative impact of poor leadership on workers and work outcomes, especially the ways that poor leadership influences these outcomes. This is true in the context of worker health and safety, particularly in the context of passive safety leadership as a form of poor leadership. Passive leadership, which has been identified as a form of laissez-faire leadership, generally ignores worker needs, ignores workplace issues or problems, and has been depicted as an absence of effective leadership [3–5]. Regarding workplace safety, this generally means leaders are not responsive to worker safety and health needs and generally react only when a safety-related incident is imminent or occurs [3,4]. This contrasts with transformational leadership approaches that motivate workers through leading by example, communicating a clear and appealing vision, actively caring for workers, and motivating followers to pursue higher-order needs [5–7].

Passive leadership can be detrimental to worker health and wellbeing [8,9] and worker safety outcomes [3,4,10,11]. Much of the research associated with poor leadership, including passive leadership, has been in the context of stress and health impairment [2,12]. However, there is a dearth of information regarding how these influences on stress and health impairment may impact safety behaviors. More research and scientific exploration in this area is warranted. As such, there is a need to examine the ways passive safety leadership influences safety behavior outcomes. This is especially true within the fire service as research with this occupational group is limited. Means to bolster safety behaviors and to curtail unsafe acts are needed within the fire service as unsafe acts, a lack of compliance with standard operating procedures, and non-participation in safety can result in significant exposures, injuries, illnesses, or even fatalities among firefighters [13–16]. Also, safe performance among firefighters is critical to ensure mission completion and to protect the public during emergency situations [17].

Research conducted by Smith, Eldridge, and DeJoy [4] provides some insights into the relationships between passive safety leadership and safety behavior outcomes among firefighters. They determined that passive leadership was negatively associated with safety climate and that safety climate positively influenced safety behavior outcomes; however, it was determined that there was no direct, significant relationship between passive leadership and safety compliance behavior and safety participation behavior [4]. The relationship was indirect and mediated through safety climate [4]. As this research focused on passive leadership and safety climate as a mediating factor, it did not include stress or other health impairment outcomes within the model or analyses. Research is warranted in this area, particularly since passive leadership has been associated with increased perceptions of stress [8,18,19] and because stress has been negatively associated with firefighter safety behavior outcomes, albeit usually indirectly and mediated by other factors such as burnout [20,21].

Relationships between passive leadership, stress, anxiety, and safety behaviors among firefighters are not well understood. Associations between work-related stress and anxiety have been illustrated in the past with workers other than firefighters [22–24]. These stressors are generally related to job demands, workload, time pressure, job control, and role clarity [22–24]. These stressors have also been linked to anxiety among firefighters [25,26]. The quality of leadership can influence these factors, especially relationships between management and firefighters. Relationship conflict is a significant predictor of anxiety among firefighters [26]. Thus, it is probable that passive leadership might produce internal relationship conflict, resulting in anxiety among firefighters.

**Figure 1.**
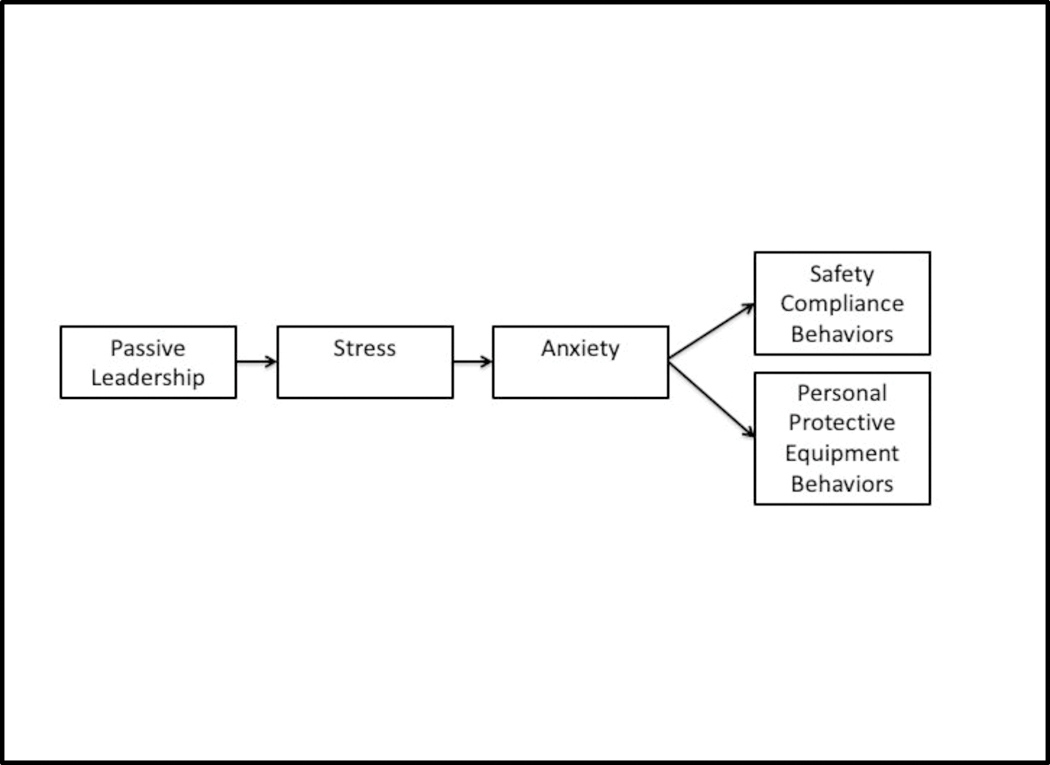
Hypothesized model.

The focus of the present research was to examine the relationships within a proposed model (see Figure 1) that includes passive leadership, stress, anxiety, and two compliance-oriented safety behavior outcomes within a large sample of career firefighters. It was hypothesized that passive leadership would be positively associated with perceptions of work stress (Hypothesis 1). Additionally, stress was predicted to be positively associated with anxiety (Hypothesis 2). In prior research, stress generally impacts safety behavior outcomes indirectly among firefighters when more distal health impairment factors are examined [20,21]. Based on this framework and because more parsimonious models are preferred when evaluating theoretical models, we hypothesized that stress influences anxiety and that anxiety would be negatively associated with safety compliance (Hypothesis 3) and personal protective equipment behavior (Hypothesis 4). In the proposed model, stress was not posited to have a direct impact on behavior outcomes.

Should the research findings support these hypotheses, this research will provide empirical evidence of these relationships, which have both practical and research implications. The findings would delineate the consequences of passive leadership in the fire service, which could ultimately impact the overall safety, health, and wellbeing of firefighters.

## 2. Materials and Methods

### 2.1. Data Collection and Participants

Cross-sectional survey data (*n* = 994) were collected via electronically administered surveys made available online to full-time, career fire service members at two metropolitan fire departments in the United States. In total, 66% of available members participated in this study (*n* = 464) at one department and 53% of available members participated in this study (*n* = 530) at the second department. For the purposes of this study, participants were limited to firefighters and company officers (*n* = 742), thusly excluding those in rank above Captain to include various levels of chiefs. Data from participants with missing data for the study variables were not included in the path analysis. The final sample was *n* = 708. A summary of participant characteristics is presented in Table 1.

Researchers obtained institutional review board approval for this study though their universities prior to initiating the study. Additional approval was obtained from the Department of Homeland Security Regulatory Compliance Office as the Federal Emergency Management Agency provided funding to support this study. Prior to starting the electronic survey, participants had to acknowledge consent, which was presented to them after accessing the link to the survey. If they agreed to participate, the survey was presented to the participant. If they declined to participate, the survey was closed.

### 2.2. Measures

Measures in the model included safety-specific passive leadership, stress, anxiety, safety compliance, and personal protective equipment behavior. These measures were scales transformed in the preliminary analyses. Safety-specific passive leadership was comprised of three items used in previous firefighter research by Smith and colleagues [4]. These items originated from a measure of safety-specific passive leadership by Kelloway and colleagues [3]. Items were assessed on a 5-point Likert-type scale with response options from strongly disagree to strongly agree. The items included “my immediate supervisor avoids making decisions that affect safety on the job”, “my immediate supervisor fails to intervene until safety problems become serious”, and “my immediate supervisor waits for things to go wrong before taking action”.

Stress was comprised of six items derived from the work of DeJoy and colleagues [27]. These items have been used to assess perceptions of work stress among firefighters [20,21]. Some of the items in the measure include “in the last month, how often have you felt nervous and stressed because of work” and “in the last month, how often have you felt you were unable to control the important things at work”. Items were assessed on a 5-point Likert-type scale with response options from almost never to almost always.

Anxiety was comprised of two items. Items were derived from an existing measure for anxiety [28]. Items included “over the past month, how often have you been bothered by feeling nervous, anxious or on edge”, and “over the past month, how often have you been bothered by not being able to stop or control worrying?” Response options included not at all, several days, more than half the days, and nearly every day.

There were two distinct compliance-oriented firefighter safety behaviors included in the model and analyses. Safety compliance was comprised of six items, which originated from Neal and Griffin [29] and were used in prior firefighter safety research [20,21]. Personal protective equipment behavior was comprised of three items derived from prior firefighter safety research [20,21]. The safety compliance measure included items such as “how often do you use the correct safety procedures for carrying your job” and “how often do you ensure the highest levels of safety when you carry out your job?” The three personal protective equipment behavior items included “I correctly use appropriate personal protective equipment (PPE) during firefighting operations”, “I correctly inspect all my PPE on a regular basis”, and “I ensure my PASS device is fully functional prior to each use”. Each of the items were assessed on a 5-point Likert-type scale with response options from almost never to almost always.

### 2.3. Analysis

Preliminary statistical analyses, including transforming items into scales; descriptive analyses; analyses of correlations; and assessment of Cronbach’s alphas were completed using SPSS (v.25). A path analysis, using Mplus (v.8.3), was completed to examine the hypothesized model and its relationships (see Figure 1). Model fit was assessed using multiple fit indices including the Root Mean Square Error of Approximation (RMSEA), the Standardized Root Mean Square Residual (SRMR), and the Comparative Fit Index (CFI). Unstandardized path coefficients, standard errors, and significance values were examined to determine if hypothesized pathways were significant.

## 3. Results

Descriptive statistics, measures of univariate normality including skewness and kurtosis, and correlations were examined, indicating data were appropriate for completing analyses. Skewness and kurtosis met levels deemed acceptable by Brown [30]. An assessment of the correlation matrix did not indicate concerns with multicollinearity. Cronbach’s alphas are generally consistent with quality research. Descriptive statistics, Cronbach’s alphas, and correlations for each of the measures are presented in Table 2.

Regarding the model analysis, the overall fit of the hypothesized model was very good: χ^2^ = 13.16, *df* = 5, *p* = 0.02, RMSEA = 0.05, SRMR = 0.02, and CFI = 0.99. As illustrated in Table 3, all paths were significant and in the hypothesized direction. Passive safety leadership was significantly, positively associated with perceptions of stress. Stress was significantly, positively associated with anxiety. Anxiety was significantly, negatively associated with safety compliance and personal protective equipment behaviors. As such, all hypotheses were supported.

In addition to testing the most parsimonious, theoretically justified model, which was hypothesized, an alternate model was tested. The fit of this model was also very good: *χ*^2^ = 9.525, *df* = 2, *p* = 0.01, RMSEA = 0.07, SRMR = 0.02, and CFI = 0.99. However, RMSEA increased. This model was not significantly different when applying a chi-square difference test (*p* = 0.30), suggesting both models fit equally well statistically.

In this alternate model, a path from passive leadership to anxiety was examined, along with the paths from passive leadership to stress and stress to anxiety. The relationship between passive leadership and anxiety was not significant (*p* = 0.063), illustrating that the relationship between passive leadership and anxiety is mediated by stress, as significance was maintained between passive leadership and stress (*p* < 0.0001) and stress and anxiety (*p* < 0.0001).

Additionally, relationships between stress and the behavior outcomes were examined in the alternate model. Stress has been presumed to have direct effects on safety behavior outcomes; however, it has been noted that these relationships are usually mediated by more distal health impairment outcomes resultant of stress. This has been the case among firefighters where burnout mediated the relationship between stress and safety behaviors [20]. This also appears to be the case in this study. It was determined that the relationships between stress and safety compliance (*p* = 0.87) and stress and personal protective equipment behaviors (*p* = 0.68) were not significant. The impact of stress in this case illustrates mediated relationships, as the relationship between stress and anxiety remained significant (*p* < 0.0001) and the relationships between anxiety and each of the behaviors remained significant (*p* < 0.0001).

## 4. Discussion

Although some research has explored direct relationships between safety-specific passive leadership and safety behaviors, little research has examined possible mediating factors of that relationship aside from safety climate, particularly in the context of fire-fighting. In this novel study, we examined relationships between safety-specific passive leadership and stress, stress and anxiety, and anxiety and two safety behavior outcomes.

This study determined that safety-specific passive leadership had a significant influence on firefighter stress perceptions. Further, stress perceptions positively influenced firefighter anxiety. Importantly, it was determined that anxiety had detrimental influences on firefighter compliance-oriented behaviors related to safety compliance and personal protective equipment behavior. Overall, the model and delineated relationships illustrate the detrimental consequences of safety-specific passive leadership among a sample of firefighters.

Passive leaders, in the context of safety, are devoid of action, are generally not actively managing risks and safety issues, and show little to no concern for worker safety, health, and wellbeing [3,4,31]. This lack of action or exhibition of concern for firefighters appears to be distressing to firefighters, which can exacerbate anxiety. These negative implications to health may be linked to views that this leadership style is not supportive. Beyond this, this form of leadership may result in feelings among firefighters that they have less control and fewer resources, which may be associated with increased stress, as suggested by the Conservation of Resources (COR) theory [32,33].

Additional research is needed to explore why firefighters are distressed when their leaders are passive in the context of safety leadership. Future research might examine the COR theory and/or other stress-related theoretical models that might support these outcomes [34], such as the Person–Environment Fit theory [35,36], the Job Demands–Control framework [37], or the Effort–Reward Imbalance theory [38], among others. Studies testing these theories might provide more insight into means to reduce distress or means to cope when leaders are perceived as passive. No matter the causal mechanism, efforts are needed to bolster support among firefighters. Perceived support among firefighters has generally been associated with less stress [39–42].

From a practical standpoint, it has been determined that safety-specific transformational leadership and associated leadership styles that promote empowerment in the fire service may provide opportunities to bolster safety outcomes, including safety behaviors and personal protective equipment use [4,43,44]. Transformational leaders, in contrast to passive leaders, focus on leading positively by example, communicate clearly, present a clear vision for their workers, empower workers, actively care for their workers, and motivate workers under their leadership to achieve higher-order needs [5–7]. Although an examination of relationships between transformational leadership and the constructs in our model was beyond the scope of the present study, it would be beneficial for researchers and administrators to learn whether transformational leadership strategies, particularly safety-specific strategies, are negatively associated with stress perceptions among firefighters. If so, efforts to bolster transformational leadership strategies may concurrently protect health and promote health and wellbeing, particularly if these strategies reduce distress and associated anxiety. In broader work populations, there is some evidence that transformational leadership is associated with worker wellbeing [45,46]. This suggests transformational leadership might serve as a targeted area for tailored interventions to bolster worker health outcomes [45]. This would likely be embraced in the fire service where the focus is often on multi-session worker training programs to address mental health instead of programs at the organizational level [47].

In conjunction with a transformational leadership intervention that could be implemented within fire service organizations, tertiary level interventions by occupational health professionals and leaders could be implemented as part of this integrated intervention program to help those with health decrements [48]. Generally, efforts to bolster mental health in public safety and the fire service are focused on primary prevention to prevent stress and health impairment among individuals. These programs often address resiliency, mindfulness, and relaxation techniques to prevent stress and health impairment [47] but are not focused on restoring health. The literature suggests that individual-level interventions, such as cognitive behavioral therapy (CBT), may be effective in restoring and promoting mental health [49,50]. Thus, these efforts may also be incorporated into a holistic, integrated approach. These interventions may not only promote the health of firefighters but may serve to protect them from injury, especially since it has been delineated that anxiety hinders safety behavior outcomes, including personal protective equipment use, which is vital to protecting firefighter safety, health, and wellbeing. Within the fire service, personal protective equipment use, storage, and maintenance are essential, particularly when it is understood that effective personal protective equipment use and compliance are low among fire service members [14,15,44,51]. This integrated approach may also provide additional enhancements in personal protective equipment behaviors beyond other methods such as training [52], design solutions [53], and other organizational strategies [44].

Integrating such a program as part of a Total Worker Health^®^ approach would be novel and would further include health professionals in the worker prevention and treatment process. Such interventions have been successful in other settings. For instance, counseling professionals applying transformational leadership strategies had greater influences on outcomes such as distress and anxiety among their clients [54].

This study should be interpreted with respect to some potential limitations. Cross-sectional data limit our ability to suggest causal inferences. However, the pathways of the model and its relationships were theoretically derived and posited a priori. Under these conditions, model testing is often considered confirmatory [55]. Regarding the sample, participants were career firefighters. Volunteer firefighters did not participate in this study. As such, generalizability to other firefighters, including volunteer firefighters, needs to be evaluated. Lastly, data collected from firefighters included self-report data. Self-report data are susceptible to common method biases [56]. Other modes of data collection were not completed to assess the proposed relationships through other means, including anxiety. Anxiety was self-reported through valid and reliable survey items versus clinical diagnoses.

The results of this research are novel and have significant implications for the fire service, for promoting firefighter health and wellbeing, and for protecting firefighters. This research also provides insights into future research needs. Future research should explore these relationships through additional methods, using more than one source of data beyond self-report data, and across time as an alternate to cross-sectional methods. It would also be beneficial to explore a similar model that includes depression as a possible mediating factor. The present study shows anxiety is a detrimental outcome of passive leadership and stress and has negative consequences on safety behaviors. Similarly, Smith and colleagues identified burnout, because of stress, had similar attributes in that burnout negatively influenced safety behavior outcomes [20,21]. The impact of depression on safety behavior outcomes has not been thoroughly evaluated. As such, researchers should explore these relationships in more depth and could theoretically include them in a similar model or framework. Lastly, as was noted above, the present study did not include volunteer firefighters. Studies including volunteer firefighters are warranted given the vast number of volunteer firefighters in the fire service. Particularly, studies related to leadership are needed given there may be different leadership strategies and tactics employed by volunteer leaders in the fire service.

## 5. Conclusions

The path analysis determined that the hypothesized model fit was very good and hypothesized relationships were confirmed. Safety-specific passive leadership was positively, significantly associated with increased firefighter stress perceptions and stress was positively, significantly associated with anxiety. Anxiety was negatively, significantly associated with both safety compliance and personal protective equipment behavior. The novel results have implications for the fire service, for promoting firefighter health and wellbeing, and for protecting firefighters, emphasizing the importance of active leadership in the fire service since passive leadership, in the context of safety, is distressing, impairs health and wellbeing, and ultimately diminishes safety behavior outcomes. These weakened behaviors, non-compliance, and inappropriate personal protective equipment use increase the risk of firefighter injury, illness, or death.

## Figures and Tables

**Table 1.** Demographic information.

	*n* (%)

**Gender**	
Male	707 (97%)
Female	25 (3%)
**Race**	
Black or African American	110 (16%)
Asian	42 (6%)
American Indian or Alaskan Native	11 (2%)
Native Hawaiian or Pacific Islander	13 (2%)
White	427 (61%)
Other	100 (14%)
**Hispanic/Latino Ethnicity**	
Yes	112 (15%)
No	612 (85%)
**Marital Status**	
Single	132 (18%)
Divorced/Separated	52 (7%)
Widowed	1 (0.1%)
Married/Living w/Partner	547 (75%)
**Education**	
Some High School	1 (0.1%)
High School Graduate or GRE	38 (5%)
Some College/Technical/Vocational Training	235 (32%)
Associate Degree	219 (30%)
Bachelor’s Degree	220 (30%)
Postgraduate Coursework or Degree	23 (3%)
**Rank**	
Firefighter	488 (67%)
Company Officer	239 (33%)
**Tenure (Years with Department)**	
Less than 1	40 (6%)
1 to 3	102 (14%)
4 to 9	179 (24%)
10 to 15	177 (24%)
16 to 20	35 (5%)
21 to 25	124 (17%)
More than 25	74 (10%)

**Table 2.** Descriptive statistics, Cronbach’s alphas, and correlation matrix for latent variables.

	Passive Leadership	Stress	Anxiety	Safety Compliance	PPE Behaviors
Items (#)	3	6	2	6	3
Cronbach’s α	0.75	0.91	0.83	0.91	0.76
Mean	2.39	1.90	1.43	4.25	4.64
SD	0.88	0.93	0.75	0.67	0.73
Skewness	0.55	1.17	2.38	−2.01	−2.94
Kurtosis	0.46	0.94	6.38	6.55	9.55
Passive Leadership	1.00				
Stress	0.14	1.00			
Anxiety	0.06	0.44	1.00		
Safety Compliance	−0.03	−0.20	−0.44	1.00	
PPE Behaviors	−0.03	−0.19	−0.44	0.60	1.00

All correlations were significant at *p* < 0.001; PPE is personal protective equipment.

**Table 3.** Path model analysis results.

Path	Unstandardized Path Coefficient	*SE*	*t*	*p*
Passive Safety Leadership → Stress	0.15	0.04	3.80	<0.001
Stress → Anxiety	0.36	0.03	13.05	<0.001
Anxiety → Safety Compliance	−0.39	0.03	−13.16	<0.001
Anxiety → PPE Behavior ^1^	−0.43	0.03	−13.97	<0.001

1 PPE is personal protective equipment.

## Data Availability

Restrictions apply to the availability of these data. Please contact the corresponding author for details and data requests.

## References

[1] Day RC; Hamblin RL Some effects of close and punitive styles of supervision. Am. J. Sociol 1964, 69, 499–510.

[2] Kelloway EK; Sivanathan N; Francis L; Barling J. Poor leadership. In Handbook of Work Stress; Barling J, Kelloway EK, Frone MR, Eds.; SAGE Publications, Inc.: Thousand Oaks, CA, USA, 2005; pp. 89–112.

[3] Kelloway EK; Mullen J; Francis L. Divergent effects of transformational and passive leadership on employee safety. J. Occup. Health Psychol 2006, 11, 76–86. 16551176 10.1037/1076-8998.11.1.76

[4] Smith TD; Eldridge F; DeJoy DM Safety-specific transformational and passive leadership influences on firefighter safety climate perceptions and safety behavior outcomes. Saf. Sci 2016, 86, 92–97.

[5] Yukl G. Leadership in Organizations, 6th ed.; Pearson Prentice Hall: Upper Saddle River, NJ, USA, 2006.

[6] Barling J; Loughlin C; Kelloway EK Development and test of a model linking safety-specific transformational leadership and occupational safety. J. Appl. Psychol 2002, 87, 488–496. 12090606 10.1037/0021-9010.87.3.488

[7] Bass BM Leadership and Performance beyond Expectations; The Free Press: New York, NY, USA, 1985.

[8] Barling J; Frone MR If Only my Leader Would just Do Something! Passive Leadership Undermines Employee Well-being Through Role Stressors and Psychological Resource Depletion. Stress Health 2017, 33, 211–222. 27470980 10.1002/smi.2697 PMC5791740

[9] Mullen J; Kelloway EK Occupational health and safety leadership. In Handbook of Occupational Health Psychology; Quick JC, Tetrick LE, Eds.; American Psychological Association: Washington, DC, USA, 2011; pp. 357–372.

[10] Jiang L; Probst TM Transformational and passive leadership as cross-level moderators of the relationships between safety knowledge, safety motivation, and safety participation. J. Saf. Res 2016, 57, 27–32. 10.1016/j.jsr.2016.03.002 27178076

[11] Mullen J; Kelloway EK; Teed M. Inconsistent style of leadership as a predictor of safety behaviour. Work. Stress 2011, 25, 41–54.

[12] Offermann LR; Hellmann PS Leadership behavior and subordinate stress: A 36000 view. J. Occup. Health Psychol. 1996, 1, 382. 9547060 10.1037//1076-8998.1.4.382

[13] Hard DL; Marsh SM; Merinar TR; Bowyer ME; Miles ST; Loflin ME; Moore PH Summary of recommendations from the National Institute for Occupational Safety and Health fire fighter fatality investigation and prevention program, 2006–2014. J. Saf. Res 2019, 68, 21–25. [PubMed] 10.1016/j.jsr.2018.10.013 PMC9273025 30876513

[14] Kahn SA; Palmieri TL; Sen S; Woods J; Gunter OL Factors implicated in safety-related firefighter fatalities. J. Burn. Care Res. 2017, 38, e83–e88. [PubMed] 27606562 10.1097/BCR.0000000000000434

[15] Kunadharaju K; Smith TD; DeJoy DM Line-of-duty deaths among U.S. firefighters: An analysis of fatality investigations. Accid. Anal. Prev 2011, 43, 1171–1180. 21376916 10.1016/j.aap.2010.12.030

[16] Smith TD; DeJoy DM Safety climate, safety behaviors and line-of-duty injuries in the fire service. Int. J. Emerg. Serv 2014, 3, 49–64.

[17] Smith TD Examination of safety climate, affective organizational commitment, and safety behavior outcomes among fire service personnel. Disaster Med. Public Health Prep. 2020, 14, 559–562. 31769378 10.1017/dmp.2019.117

[18] Che XX; Zhou ZE; Kessler SR; Spector PE Stressors beget stressors: The effect of passive leadership on employee health through workload and work–family conflict. Work. Stress 2017, 31, 338–354.

[19] Diebig M; Bormann KC The dynamic relationship between laissez-faire leadership and day-level stress: A role theory perspective. Ger. J. Hum. Resour. Manag 2020, 34, 324–344.

[20] Smith TD; Hughes K; DeJoy DM; Dyal M-A Assessment of relationships between work stress, work-family conflict, burnout and firefighter safety behavior outcomes. Saf. Sci 2018, 103, 287–292.

[21] Smith TD; Mullins-Jaime C; Dyal MA; DeJoy DM Stress, burnout and diminished safety behaviors: An argument for Total Worker Health^®^ approaches in the fire service. J. Safety Res. 2020, 75, 189–195. 33334477 10.1016/j.jsr.2020.09.010 PMC8509082

[22] Cherry N. Stress, anxiety and work: A longitudinal study. J. Occup. Psychol 1978, 51, 259–270.

[23] Ganster DC; Rosen CC Work stress and employee health: A multidisciplinary review. J. Mgt 2013, 39, 1085–1122.

[24] Melchior M; Caspi A; Milne BJ; Danese A; Poulton R; Moffitt TE Work stress precipitates depression and anxiety in young, working women and men. Psychol. Med 2007, 37, 1119–1129. 17407618 10.1017/S0033291707000414 PMC2062493

[25] Lourel M; Abdellaoui S; Chevaleyre S; Paltrier M; Gana K. Relationships between psychological job demands, job control and burnout among firefighters. N. Am. J. Psychol 2008, 10, 489–496.

[26] Payne N; Kinman G. Job demands, resources and work-related well-being in UK firefighters. Occup. Med 2019, 69, 604–609. 10.1093/occmed/kqz167 31925427

[27] DeJoy DM; Wilson MG; Vandenberg RJ; McGrath-Higgins AL; Griffin-Blake CS Assessing the impact of healthy work organization intervention. J. Occup. Organ. Psychol 2010, 83, 139–165.

[28] Connor M; Marc Framer E; Umland B; Anderson D; Alexander G; Brennan M; Flynn J; Grossmeier J; Hamlin B; Juster IA; Program measurement & evaluation guide: Core metrics for employee health management. Am. J. Health Promot. 2014, 28, TAHP-2–TAHP-10. 24719905

[29] Neal A; Griffin MA A study of the lagged relationships among safety climate, safety motivation, safety behavior, and accidents at the individual and group levels. J. Appl. Psychol 2006, 91, 946. 16834517 10.1037/0021-9010.91.4.946

[30] Brown TA Confirmatory Factor Analysis for Applied Research; The Guilford Press: New York, NY, USA, 2006.

[31] Zohar D. The effects of leadership dimensions, safety climate, and assigned priorities on minor injuries in work groups. J. Organ. Behav 2002, 23, 75–92.

[32] Hobfoll SE Conservation of resources: A new attempt at conceptualizing stress. Am. Psychol 1989, 44, 513. 2648906 10.1037//0003-066x.44.3.513

[33] Hobfoll SE The influence of culture, community, and the nested-self in the stress process: Advancing conservation of resources theory. Appl. Psychol 2001, 50, 337–421.

[34] Ganster DC; Perrewé PL Theories of occupational stress. In Handbook of Occupational Health Psychology; Quick JC, Tetrick LE, Eds.; American Psychological Association: Washington, DC, USA, 2011; pp. 37–53.

[35] Lofquist LH; Dawis RV Adjustment to Work: A Psychological View of Man’s Problems in a Work-Oriented Society; Appleton-CenturyCrofts: New York, NY, USA, 1969.

[36] Caplan RD; Cobb S; French JRP; Van Harrison R; Pinneau SR Job Demands and Worker Health: Main Effects and Occupational Differences; Publication No. NIOSH 75–160; US Department of Health, Education, and Welfare: Washington, DC, USA, 1975.

[37] Karasek RA Jr. Job demands, job decision latitude, and mental strain: Implications for job redesign. Adm. Sci. Q 1979, 24, 285–308.

[38] Siegrist J. A theory of occupational stress. In Stress in the Workplace: Past, Present and Future; Dunham J, Ed.; Whurr Publishers: London, UK, 2001; pp. 52–66.

[39] Cowman SE; Ferrari JR; Liao-Troth M. Mediating effects of social support on firefighters’ sense of community and perceptions of care. J. Community Psychol. 2004, 32, 121–126.

[40] Oginska-Bulik N. The role of personal and social resources in preventing adverse health outcomes in employees of uniformed professions. Int. J. Occup. Med. Environ. Health 2005, 18, 233–240. 16411561

[41] Regehr C; Hill J; Knott T; Sault B. Social support, self-efficacy and trauma in new recruits and experienced firefighters. Stress Health 2003, 19, 189–193.

[42] Varvel SJ; He Y; Shannon JK; Tager D; Bledman RA; Chaichanasakul A; Mendoza MM; Mallinckrodt B. Multidimensional, threshold effects of social support in firefighters: Is more support invariably better? J. Couns. Psychol 2007, 54, 458.

[43] Smith TD; DeJoy DM; Dyal MA Safety specific transformational leadership, safety motivation and personal protective equipment use among firefighters. Saf. Sci 2020, 131, 104930. 10.1016/j.ssci.2020.104930 PMC8489483 34611382

[44] Maglio MA; Scott C; Davis AL; Allend J; Taylor JA Situational pressures that influence firefighters’ decision making about personal protective equipment: A qualitative analysis. Am. J. Health Behav 2016, 40, 555–567. 27561858 10.5993/AJHB.40.5.2

[45] Schmidt B; Loerbroks A; Herr R; Litaker D; Wilson M; Kastner M; Fischer J. Psychosocial resources and the relationship between transformational leadership and employees’ psychological strain. Work 2014, 49, 315–324. 24004772 10.3233/WOR-131713

[46] Arnold KA Transformational leadership and employee psychological well-being: A review and directions for future research. J. Occup. Health Psychol 2017, 22, 381. 28150998 10.1037/ocp0000062

[47] Edgelow M; Scholefield E; McPherson M; Mehta S; Ortlieb A. A review of workplace mental health interventions and their implementation in public safety organizations. Int. Arch. Occup. Environ. Health 2021, 95, 645–664. 34628523 10.1007/s00420-021-01772-1

[48] Tetrick LE; Quick JC Overview of occupational health psychology: Public health in occupational settings. In Handbook of Occupational Health Psychology; Quick JC, Tetrick LE, Eds.; American Psychological Association: Washington, DC, USA, 2011; pp. 3–20.

[49] Bhui KS; Dinos S; Stansfeld SA; White PD A synthesis of the evidence for managing stress at work: A review of the reviews reporting on anxiety, depression, and absenteeism. J. Environ. Public Health 2012, 2012, 515874. 10.1155/2012/515874 PMC3306941 22496705

[50] Modini M; Abbott MJ; Hunt C. A systematic review of the psychometric properties of trait social anxiety self-report measures. J. Psychopathol. Behav. Assess 2015, 37, 645–662.

[51] Kahn SA; Woods J; Sipes JC; Toscano N; Bell DE Firefighter safety: Rampant unsafe practices as documented in mainstream media. J. Burn Care Res 2014, 35, 426–430. 25106028 10.1097/BCR.0000000000000016

[52] Park HS; Ham S; Jeong JH; Kim SJ; Woo H. Examination of Factors Influencing SCBA Washing Behavior among Firefighters in Metropolitan. Int. J. Environ. Res. Public Health 2022, 19, 2240. 35206426 10.3390/ijerph19042240 PMC8872399

[53] Park H; Park J; Lin S-H; Boorady LM Assessment of Firefighters’ needs for personal protective equipment. Fash. Text 2014, 1, 8.

[54] Jacob CJ; Stoler J; Roth G. A pilot study of transformational leadership and college counseling outcomes. J. Creat. Ment. Health 2017, 12, 180–191.

[55] Kline R. Principles and Practice of Structural Equation Modeling; Guilford Press: New York, NY, USA, 2005.

[56] Podsakoff PM; MacKenzie SB; Lee J-Y; Podsakoff NP Common method biases in behavioral research: A critical review of the literature and recommended remedies. J. Appl. Psychol 2003, 88, 879. 14516251 10.1037/0021-9010.88.5.879

